# Melatonin Can Strengthen the Effect of Retinoic Acid in HL-60 Cells

**DOI:** 10.3390/ijms19102873

**Published:** 2018-09-21

**Authors:** Olga Krestinina, Roman Fadeev, Alexey Lomovsky, Yulia Baburina, Margarita Kobyakova, Vladimir Akatov

**Affiliations:** 1 Institute of Theoretical and Experimental Biophysics, Russian Academy of Science, Institutskaya St, 3, 142290 Pushchino, Moscow region, Russia; fadeevrs@gmail.com (R.F.); lomovskyalex@gmail.com (A.L.); byul@rambler.ru (Y.B.); ritaaaaa49@gmail.com (M.K.); akatov.vladimir@gmail.com (V.A.); 2 Pushchino State Natural Science Institute, 142290 Pushchino, Moscow region, Russia

**Keywords:** acute promyelocytic leukemia, HL-60 cells, melatonin, apoptosis, retinoic acid, voltage dependent anion channel-1, translocator protein, 2′,3′-cyclonucleotide-3′-phosphodiesterase

## Abstract

Melatonin is produced by the pineal gland. It can be regarded as an anticancer agent and used for combined therapy, owing to its oncostatic, antioxidant, and immunoregulatory activities. Retinoic acid is widely used for the treatment of acute promyelocytic leukemia; however, it has adverse effects on the human organism. We investigated the effect of melatonin and reduced concentrations of retinoic acid on the activation of proliferation in acute promyelocytic leukemiaon a cell model HL-60. The combined effect of these compounds leads to a reduction in the number of cells by 70% and the index of mitotic activity by 64%. Combined treatment with melatonin and retinoic acid decreased the expression of the Bcl-2. The mitochondrial isoform VDAC1 can be a target in the treatment of different tumors. The combined effect of and retinoic acid at a low concentration (10 nM) decreased VDAC1 expression. Melatonin in combination with retinoic acid produced a similar effect on the expression of the translocator protein. The coprecipitation of VDAC with 2′,3′-cyclonucleotide-3′-phosphodiesterase implies a possible role of its in cancer development. The combined effect of retinoic acid and melatonin decreased the activity of the electron transport chain complexes. The changes in the activation of proliferation in HL-60 cells, the mitotic index, and Bcl-2 expression under combined effect of retinoic acid (10 nM) with melatonin (1 mM) are similar to changes that are induced by 1 μM retinoic acid. Our results suggest that MEL is able to improve the action the other chemotherapeutic agent.

## 1. Introduction

The oncological diseases are a high-priority problem in the industrial and developing countries. New approaches to the treatment of malignant tumors are one of the most pressing problems of medicine. Therefore, the research in this area should be aimed at finding new drugs and/or combining the already known drugs to prevent the growth and development of malignant tumors. Melatonin (MEL), *N*-acetyl-5-methoxytryptamine, a hormone, is a derivative of the biogenic amine serotonin, which, in turn, is synthesized from tryptophan. MEL is secreted by the pineal gland and other tissues [1,2]. It has been reported that MEL produces an anticancer effect in different types of tumor cells [3,4]. The abnormal levels of MEL in cancer patients indicate its significant role in oncogenesis [5].

In response to oxidative stress, or when the mitochondrial matrix is overloaded by calcium, permeability of the inner membrane of mitochondria increases, and a nonspecific pore (mPTP) forms [6]. The regulators/modulators of mPTP are the voltage-dependent anion channel (VDAC), adenine nucleotide translocase, the translocator protein (TSPO), and other proteins. VDAC is a 32 kDa protein comprising three isoforms in human (VDAC1, VDAC2, VDAC3). VDAC1 is the most abundant isoform [7]. Mathupala et al. showed that some tumor cells exhibit a high level of VDAC1 expression, and the protein can be considered to be an anticancer target [8].

TSPO is a partner of VDAC. In various types of cancers, including large bowel [9], brain [10], mammary gland [11], ovary [12,13], and liver [14], the expression of TSPO is increased.

In addition, some investigators believe that TSPO plays a key role in cancer cell growth [15,16,17,18]. We have recently identified a protein (2′,3′-cyclonucleotide-3′-phosphodiesterase (CNPase)) in rat brain and liver mitochondria, namely, in mitochondrial membrane fractions, which can regulate the mPTP function [19]. Later, Yang et al. presented morphological and molecular evidence of the enhanced expression of CNPase in activated microglia [20]. Microglial cells are activated in response to pathological processes to protect the brain against damage [21,22], and CNPase is probably involved in these processes.

Acute promyelocytic leukemia (APL) is a variety of acute myelocytic leukemia, which is mainly characterized by the chromosomal translocation of the gene of the retinoic acid receptor-alpha [23]. Koh et al. showed that MEL enhances cytotoxicity, PARP (poly ADP-ribose polymerase) cleavage, and the activation of caspase and AMPKα (5′-adenosine monophosphate-activated kinase alpha) and suppresses the expression of antiapoptotic proteins, such as Bcl-2 and Bcl_xL_ in puromycin-treated HL-60 cells. The authors concluded that MEL might be useful as a sensitizer for chemotherapeutic agents, such as puromycin in APL treatment [24]. The action of MEL described in their work is consistent with the results of the other study, which shows that MEL enhances the efficiency of other chemotherapeutic drugs [25]. Combinatorial therapy with all-*trans* retinoic acid (ATRA) and conventional chemotherapeutic agents was applied as a potentially useful therapeutic approach to the treatment of APL [26,27]; however, eventual relapses during the long-term treatment diminished their therapeutic effect [28].

In the present work, the combined effect of MEL (at a pharmacological concentration—1 mM) and ATRA at a low concentration on the activation of proliferation in HL-60 cells as a model of APL was investigated, and an analysis of the cell cycle in these cells was performed. In addition, we analyzed the alterations in the content of proteins (TSPO, VDAC1, CNPase) and the basic subunits of electron transport chain (ETC) complexes by the action of MEL in combination with ATRA in these cells.

## 2. Results

**Figure 1:**
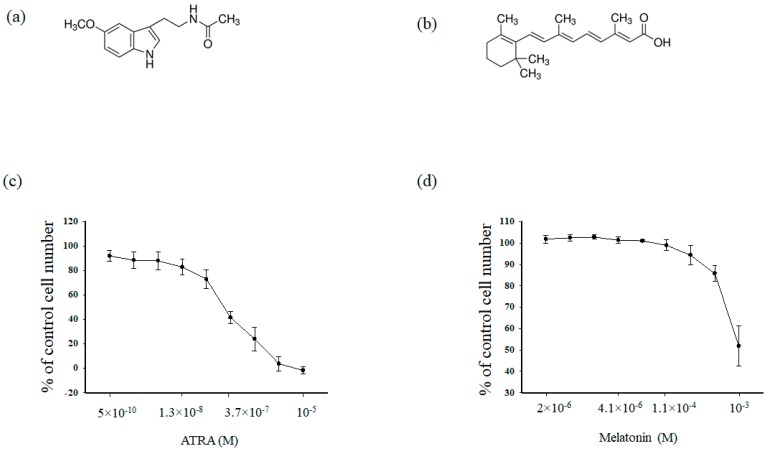
Upper part (**a**) and (**b**). Chemical structures of melatonin (MEL) and all-trans retinoic acid (ATRA). (**a**) MEL, (**b**) ATRA. Lower part (**c**) and (**d**). Concentration dependence of the cytotoxic effects of MEL and ATRA. Cells were seeded in a 96-well plate at a density of 5 × 10^3^ cells per well and treated with indicated concentrations of (**a**) MEL and (**b**) ATRA for 96 h. The data are presented as means ± S.D. of ten separate experiments.

At first, we analyzed the cytotoxic effects of MEL (Figure 1a) and ATRA (Figure 1b) in HL-60 human leukemic cells. Cells were treated for 96 h with different concentrations of either MEL (10^−3^, 3.3 × 10^−4^, 1.1 × 10^−4^, 4 × 10^−5^, 10^−5^, 4.1 × 10^−6^, 1.4 × 10^−5^, 5 × 10^−6^, 2 × 10^−6^ M) and ATRA (5 × 10^−10^, 4 × 10^−9^, 1.3 × 10^−8^, 4 × 10^−8^, 1.2 × 10^−8^, 3.77 × 10^−7^, 1.11 × 10^−5^, 3.33 × 10^−5^, 10^−5^ M) for 96 h. In clinical practice, ATRA is used at a concentration of 1 μM; in the present work, the ATRA concentration was reduced to 10 nM (Figure 1c)., MEL had a significant effect on the viability of HL-60 cells up to the concentration of 1 mM (as shown in Figure 1d).

**Figure 2:**
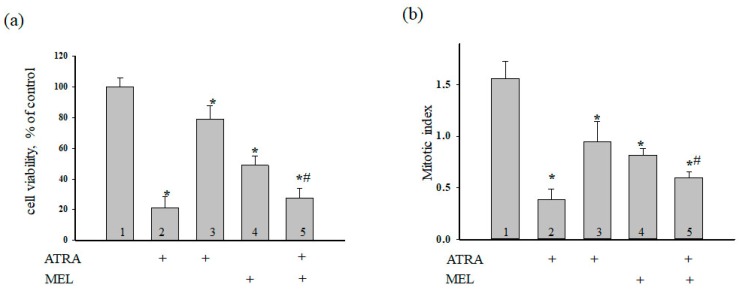
Combined effect of MEL and ATRA on the viability and proliferation status of HL-60 cells. Cells were seeded in a 96-well plate at a density of 5 × 10^3^ cells per well and treated with 1 µM ATRA (column 2), and 10 nM ATRA (columns 3) and 1 mM MEL (column 4), and MEL in combination with 10 nM ATRA (column 5); untreated cells (control, column 1). (**a**) Cell viability in % relative to the control; (**b**) The mitotic index (MI) is defined as the ratio between the number of cells in mitosis and the total number of cells. The data are presented as the means ± S.D. of ten separate experiments. * *p* < 0.05 significant difference in values in comparison with the corresponding control, ^#^ *p* < 0.05 significant difference in values compared to the value obtained after the addition of MEL alone.

Next, we evaluated the effect of MEL on cell death in HL-60 cells treated with ATRA (10 nM) and MEL at noncytotoxic concentrations (1 mM) for 96 h (Figure 2). Figure 2a demonstrates the viability of HL-60 cells under different conditions. The relative values of the number of cells are shown in percent. It is seen that the cell viability was reduced in the presence of MEL (1 mM) and ATRA (10 nM) by 50% and 20%, respectively, when compared to the control (100%). The combined effects of the compounds (ATRA + MEL) led to a 70% reduction in the number of cells as compared to the control and a 43% reduction when compared to the experiments with MEL alone. In this process, MEL significant strengthens the effect of ATRA in comparison with control (ATRA 10 nM).

The mitotic index (MI) is a measure of the proliferation status of a cell population. It is defined as the ratio between the number of cells in mitosis and the total number of cells. Here, we calculated MI of HL-60 cells under different conditions. Figure 2b demonstrates the effect of the compounds on the mitotic activity of cells. It is seen that MI decreased by ~50% in the presence of 1 mM MEL and by ~37% in the presence of 10 nM ATRA. With the combined effect of the compounds, MI was reduced by 64% as compared with the control (without treatment) and by 28% when compared to experiments with MEL alone. These results imply that MEL can enhance the cytotoxicity of ATRA at low concentration in HL-60 human leukemic cells.

**Figure 3:**
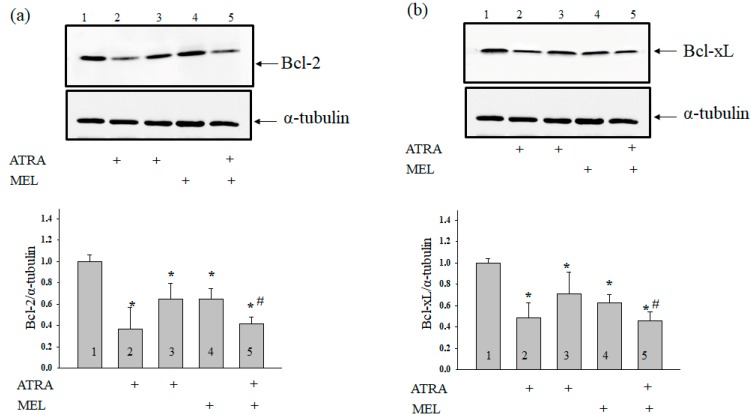
Combined effect of MEL and ATRA on the level of Bcl-2 (**a**) and Bcl-xL (**b**) proteins in HL-60 cells. Cells were seeded in a 96-well plate at a density of 5 × 10^3^ cells per well and treated with 1 µM ATRA (column 2), and 10 nM ATRA (columns 3) and 1 mM MEL (column 4), and MEL in combination with 10 nM ATRA (column 5); untreated cells (control, column 1). Protein samples were extracted and subjected to Western blot for Bcl-2 and Bcl-xL detection. Immunodetection of α-tubulin was used as a loading control. Upper parts-immunostaining of Bcl-2, Bcl-xL and α-tubulin. Lower part-quantitation of immunostaining using computer-assisted densitometry. Bar graphs represent the proteins levels in relative units. The protein level in a cell lysate without any addition was taken to be unity and served as a control. The data are presented as the means ± S.D. of five separate experiments. * *p* < 0.05 significant difference in the protein level compared with the corresponding control, ^#^ *p* < 0.05 significant difference in the protein level compared to the value that was obtained in the presence of MEL alone.

Earlier Quintana and coworkers have reported that MEL modulated the induction of apoptosis in hyperthermia-exposed human leukemia cells (U937) [29]. Here, we determined the level of Bcl-2 in our experimental conditions. Figure 3 (upper panel) shows Western blots of Bcl-2 in the lysates of HL-60 human leukemic cells treated with ATRA (1 µM), ATRA (10 nM), MEL (1 mM), and ATRA (10 nM) + MEL (1 mM). A quantitative analysis of the Bcl-2 and Bcl-xL levels are shown in Figure 3 (lower panels). Protein bands were quantified after normalization with respect to α-tubulin. The Bcl-2 level in HL-60 cells in the presence of ATRA (1 µM) was three times lower, and in the presence of ATRA (10 nM), it was two times lower than in the control. A similar result was obtained after treatment with MEL (1 mM). Interestingly, with the combined effect of MEL and ATRA, 1 mM MEL strengthened the influence of 10 nM ATRA, and the level of antiapoptotic Bcl-2 was reduced approximately threefold when compared with control and by 37% compared to experiments with MEL alone. The same effects of MEL and ATRA and its combined effect on Bcl-xL level were observed.

**Figure 4:**
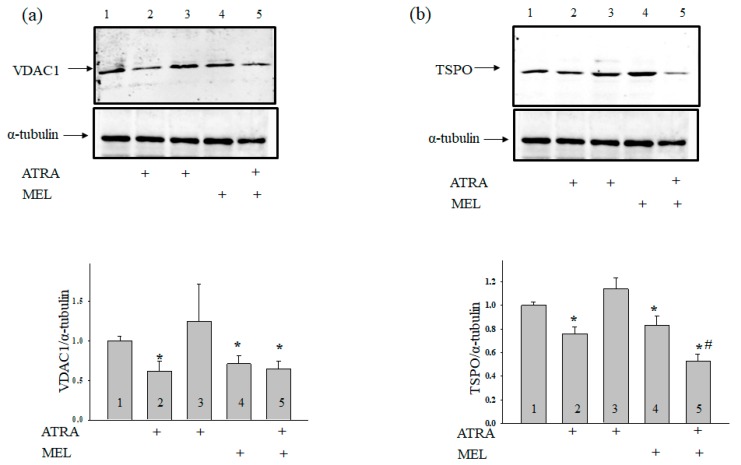
Combined effect of MEL and ATRA on the level of TSPO and VDAC1 in HL-60 cells. Cells were seeded in a 96-well plate at a density of 5 × 10^3^ cells per well and treated with 1 µM ATRA (column 2), and 10 nM ATRA (columns 3) and 1 mM MEL (column 4), and MEL in combination with 10 nM ATRA (column 5); untreated cells (control, column 1). Immunodetection of α-tubulin was used as a loading control. (**a**) Immunostaining (upper part) and quantitation (lower part) of the protein level of VDAC1; (**b**)-of TSPO. Bar graphs represent the levels of appropriate proteins in relative units. The protein level in a cell lysate without any addition was taken to be unity and served as a control. The data are presented as means ± S.D. of five separate experiments. * *p* < 0.05 significant difference in protein level in comparison with corresponding control, ^#^ *p* < 0.05 significant difference in the protein level compared to the value obtained in the presence of MEL alone.

Since VDAC initiates apoptotic-signaling cascades [30] and VDAC1 is an anticancer target [8], we determined the VDAC1 content in our experimental conditions (Figure 4a). In cells that were treated with 1 µM ATRA, the VDAC1 level was reduced by 50%, while 10 nM ATRA did not affect VDAC1 expression compared with the control. Treatment with MEL diminished the level of VDAC1 by ~30%, but the addition of MEL to cells that were treated with ATRA (10 nM) decreased the expression of VDAC1 by ~30%. Because VDAC is tightly associated with TSPO [31], we determined whether the TSPO level changes under the experimental conditions used. Figure 4b demonstrates that, in the presence of ATRA (1 µM) and MEL, the TSPO content decreased by ~24% and ~16%, respectively, while ATRA (10 nM) did not change the TSPO level in comparison with the control. The addition of MEL in combination with ATRA (10 nM) to HL-60 cells led to a ~40% decline in TSPO expression as compared with control and to a 36% decrease when compared to experiments with MEL alone. We have reported earlier that VDAC coprecipitates with CNPase [32].

**Figure 5:**
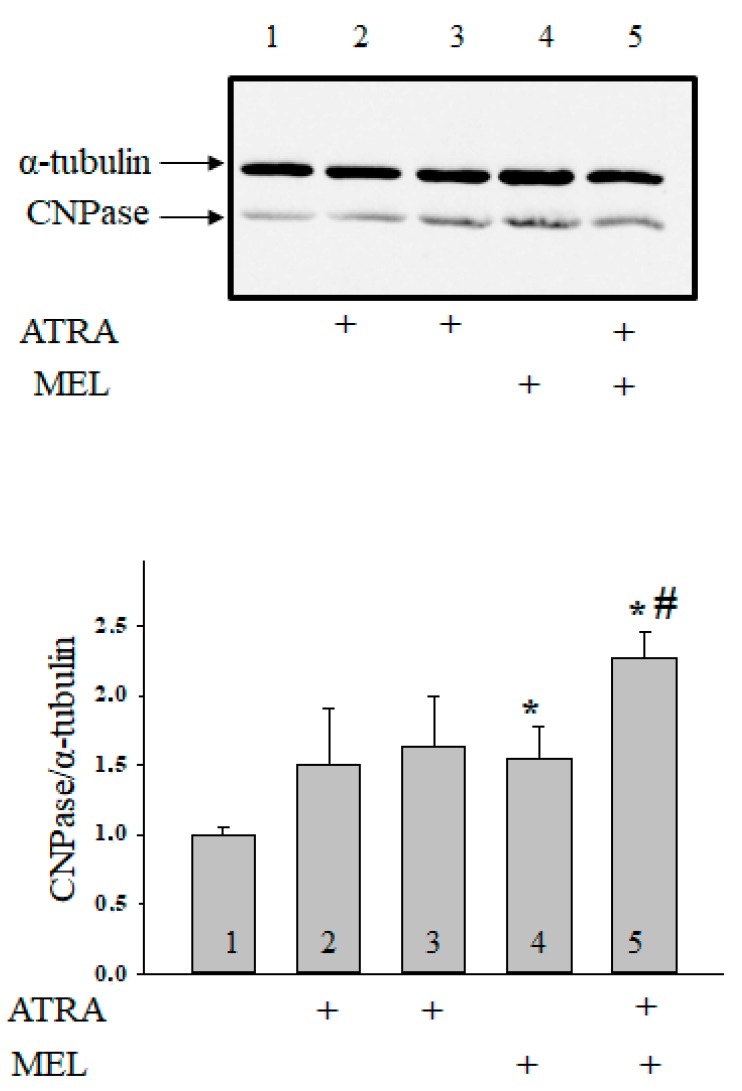
Combined effect of MEL and ATRA on the level of CNPase in HL-60 cells. Cells were seeded in a 96-well plate at a density of 5 × 10^3^ cells per well and treated with 1 µM ATRA (column 2), and 10 nM ATRA (columns 3) and 1 mM MEL (column 4), and MEL in combination with 10 nM ATRA (column 5); untreated cells (control, column 1). Protein samples were extracted and subjected to Western blot for CNPase detection. The immunodetection of α-tubulin was used as a loading control. Upper part-immunostaining of CNPase and α-tubulin. Lower part-quantitation of immunostaining using computer-assisted densitometry. Bar graphs represent the Bcl-2 level in relative units. The protein level in a cell lysate without any addition was taken to be unity and served as a control. The data are presented as means ± S.D. of five separate experiments. * *p* < 0.05 significant difference in protein level in comparison with corresponding control, ^#^ *p* < 0.05 significant difference in the protein level compared to the value obtained in the presence of MEL alone.

**Figure 6:**
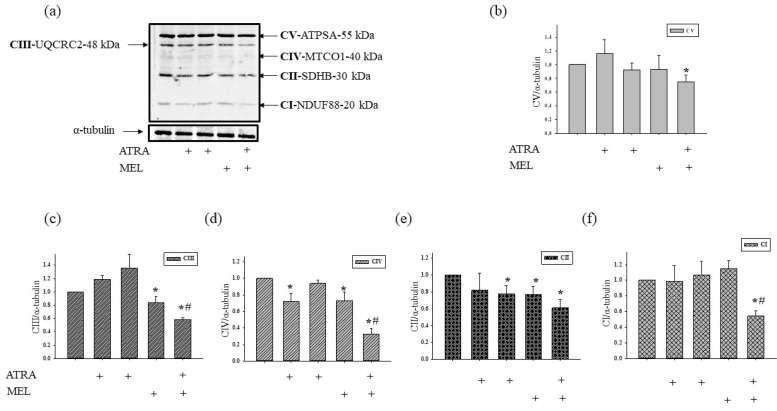
The effect of MEL and ATRA on the mitochondrial respiratory chain complexes in HL-60 cells. Cells were seeded in a 96-well plate at a density of 5 × 10^3^ cells per well and treated with 1 µM ATRA (column 2), and 10 nM ATRA (columns 3) and 1 mM MEL (column 4), and MEL in combination with 10 nM ATRA (column 5); untreated cells (control, column 1). Protein samples were extracted and subjected to Western blotting. Changes in mitochondrial complexes were detected using, the Total OXPHOS Rodent WB Antibody Cocktail. The immunodetection of α-tubulin was used as a loading control. (**a**) Immunostaining with OXPHOS antibody cocktail and α-tubulin; (**b**–**f**)-quantification of immunostaining using computer-assisted densitometry. Bar graphs represent the levels of appropriate complexes (I–V) in relative units. The protein level in a cell lysate without any addition was taken to be unity and served as a control. The data are presented as means ± S.D. of five separate experiments. * *p* < 0.05 significant difference in protein level in comparison with the corresponding control, ^#^ *p* < 0.05 significant difference in the protein level compared to the value obtained in the presence of MEL alone.

Moreover, we have found that MEL is capable of retain CNPase inside mitochondria to protect cells against damage [33]. In the present work, we determined whether the combined application of MEL and ATRA affects the CNPase level (Figure 5). ATRA at different concentrations did not change the CNPase content in HL-60 cells in comparison with the control. MEL increased the CNPase level by 55% in comparison with the control. MEL, in combination with ATRA (10 nM), upregulated the CNPase content about twofold when compared with control and by 32% as compared to experiments with MEL alone. Acuna-Castroviejo D. and co-worker showed that MEL improved the activity of ETC, reducing the formation of ROS at the level of complexes I and IV [34]. We analyzed the alterations in the content of the basic subunits of ETC complexes by the action of MEL in combination with ATRA in HL-60 cells (Figure 6). We found that the level of alpha subunit of Complex V decreased after the combined treatment with ATRA and MEL by 25%. MEL diminished the content of the Core protein 2 of Complex III by 16%; however, the addition of MEL strengthened the effect of ATRA (10 nM) with the result that the level of the Core protein 2 decreased by 32% when compared with control and by 31% as compared to experiments with MEL alone. We noticed a decline in the level of the subunit of Complex IV by 27% in the presence of ATRA (1 µM), by 26% in the presence of MEL, and by 65% with the combined effect of ATRA (10 nM) and MEL compared with control. However, the level of the subunit of Complex IV in the case of ATRA (10 nM) combined with MEL decreased by 56% ascompared to experiments with MEL alone. The level of the subunit of Complex II decreased in the presence of ATRA (10 nM), MEL, and a combination of ATRA (10 nM) with MEL by 22, 23, and 40%, respectively. After the exposure of cells to a combination of 10 nM ATRA with MEL, the content of the subunit of Complex I declined by 46% as compared with the control and by 53% when compared to experiments with MEL alone.

## 3. Discussion

The combination therapy using ATRA and chemotherapeutic drugs has significantly increased the remission and the disease-free survival rates [35,36], the harmful effects of ATRA (e.g., ATRA syndrome) and the toxicity of chemotherapeutic agents still remain urgent problems in the treatment of APL [37,38]. MEL produces a beneficial effect on different types of cancers, including pancreatic, liver, and prostate cancer cells [39,40,41,42]. It is known that MEL induces apoptosis in HL-60 cells [43,44]. In addition, MEL enhances the efficiency of other chemotherapeutic drugs [24]; however, the information on the combination therapy of MEL with ATRA is very scarce. Here, we investigated the effect of MEL in combination with ATRA at a concentration that is lower than used in medicinal practice (10 nM) on the activation of proliferation in HL-60 cells. We found that 1 µM ATRA exhibits a substantial cytotoxicity against HL-60 cells, while a concentration of 10 nM does not influence the cell viability. MEL reduced the cell viability; however, the combined treatment of MEL (1 mM) and ATRA (10 nM) at nontoxic concentrations enhanced the cytotoxicity against HL-60 cells as compared to the treatment with MEL or ATRA (10 nM) taken alone. The changes in the activation of proliferation in HL-60 cells and the mitotic index under combined effect of ATRA (10 nM) with MEL (1 mM) are similar to those that were observed by the influence of 1 μM ATRA. Furthermore, the combined treatment of MEL with ATRA significantly suppressed the expression of the antiapoptotic protein Bcl-2 as compared to the expression of the protein after the treatment with ATRA (10 nM) or MEL alone. The changes in the expression of Bcl-2 under the combined effect of ATPA (10 nM) with MEL were similar to those occurring in the presence of ATPA (1 µM). Overexpression of Bcl-2 family proteins CED-9 and Bcl-xL can induce mitochondrial fusion in mammalian cells [45]. Bcl-xL can also accelerate mitochondrial fission in mammalian neurons, thereby accelerating mitochondrial dynamics [46]. It is possible that a decrease in the level of Bcl-2 by the action of MEL, ATRA, and a combination of MEL with ATRA triggers the apoptosis signaling cascade. The regulation of the cellular apoptosis via the mitochondrial permeability transition pore (mPTP) is the balance between pro- and anti-apoptotic Bcl-2 family proteins [47]. The initial stage of apoptosis is the formation of mPTP in the inner mitochondrial membrane, which is accompanied by a membrane potential drop, mitochondrial swelling, and release of cytochrome *c* from the intermembrane space to the cytosol via the permeabilization of the outer mitochondrial membrane [48]. Molecular interactions of VDAC with pro- and antiapoptotic proteins of the outer mitochondrial membrane are multi-faceted and they can both promote and prevent cell death. The mitochondrial VDAC is a basic component that can be targeted in tumors. VDAC initiates apoptotic signaling cascades and hence is capable of depleting the metabolic fluxes of tumors [30]. We found that the expression of VDAC1 in HL-60 cells decreased after the combined treatment with ATRA (10 nM) and MEL, indicating changes in the regulation of metabolic and energetic functions of mitochondria and in the fate of cancer cells. The changes in the expression of VDAC1 under the combined effect of ATPA (10 nM) with MEL were similar to those that were occurring in the presence of ATPA (1 µM). TSPO is not only a partner but also a modulator of VDAC [49]. It has been established that TSPO is involved in the regulation of cellular proliferation and apoptosis in gliomas, which are highly aggressive malignant cancers with a poor prognosis [50]. TSPO is colocolized with VDAC and forms a firmly complex with this protein. It might modulate VDAC conductance and regulate mitochondrial functions under different physiological and pathophysiologic conditions [31].

Many investigators reported that TSPO expression increases in different types of cancer, including brain tumor and glioma [51,52,53]. The overexpression of TSPO correlates with tumorogenicity [54]. We observed a diminished TSPO expression in HL-60 cells that were treated with ATRA (1 µM), MEL, and ATRA (10 nM) combined with MEL. Presumably, the decrease in TSPO expression under these conditions causes a reduction in tumorogenicity.

In the outer membrane of mitochondria, VDAC1 is a protein that interacts with cytosol, endoplasmic reticulum, and mitochondrial proteins. Together, they regulate signaling and metabolic pathways, triggering cell death [55]. Recently, we found that VDAC co-precipitated with CNPase localized in both the outer and inner membrane [19,32]. We showed that CNPase can participate in the regulation of mPTP functioning [19]. It is known that CNPase expression in activated microglia can be upregulated in response to brain injury [20]. It remains unknown whether CNPase is involved in cancer development. There is evidence indicating that a decrease in TSPO expression is accompanied by an increase in the level of CNPase in mitochondria that are isolated from glioma C6 with TSPO knockdown [in press]. These data agree with the results obtained in the present study, which indicate that 1 µM ATRA and 10 nM ATRA combined with MEL increase CNPase expression, while the expression of TSPO in these conditions decreases. Our results suppose a possible involvement of CNPase in cancer development. Further research is needed to establish the mechanisms by which CNPase is involved in cancer progress.

Recently, we showed that CNPase is associated with ETC complexes [32]. MEL is able to improve the activity of the ETC, thereby increasing the activity of the CI and the CIV of the mitochondrial ETC in a time-dependent manner [34,56]. High activity of mitochondrial complexes is necessary for the normal functioning of cells. In our case, the activity of ETC complexes in HL-60 cells should be reduced to prevent tumor development. The combined influence of ATRA (10 nM) with MEL decreased the expression of the subunits of ETC complexes, and hence their activity.

In summary, MEL in combination with retinoic acid (10 nM) increased the cytotoxicity of ATRA toward HL-60 cells and suppressed the expression of antiapoptotic Bcl-2 protein. The changes in the activation of proliferation in HL-60 cells, the mitotic index, and Bcl-2 expression under combined effect of ATRA (10 nM) with MEL (1 mM) are similar to changes that are induced by 1 μM ATRA. The expression of VDAC1 and TSPO decreased in these conditions, whereas the CNPase expression was enhanced. We suppose that these proteins participate in cancer development. Moreover, MEL in combination with retinoic acid decreased the expression of the subunits of complexes, thereby reducing their activity. Overall, our findings suggest that MEL is capable of enhancing the action of other chemotherapeutic agents and can be used in novel strategies in cancer therapy.

## 4. Materials and Methods

### 4.1. Chemicals and Reagents

MEL (*N*-acetyl-5-methoxytryptamine), resazurin sodium salt, propidium iodide (PI), and a protease/phosphatase inhibitor cocktail were purchased from Sigma-Aldrich (St. Louis, MO, USA). The enhanced chemiluminescence (ECL) detection reagent was purchased from Bio-Rad (Hercules, CA, USA). MEL was dissolved in ethanol.

### 4.2. Cell Culture

Human promyelocytic leukemia HL-60 cells (CCL-240) were derived from ATCC (Manassas, VA, USA). Cells were grown in RPMI 1640 medium (Sigma-Aldrich, USA) supplemented with 20% heat-inactivated fetal bovine serum (Gibco, Grand Island, NY, USA) and 40 µg/mL of gentamycin sulfate (Sigma-Aldrich, USA) at 37 °C under conditions of 95% air humidity and 5% CO_2_. The cultures exhibited characteristic doubling times of approximately 24 h.

### 4.3. Cell Viability Assay

The viability of cells was evaluated while using the resazurin cell viability assay. In brief, cells were seeded in a 96-well plate at a density of 5 × 10^3^ cells per well. After 24 h, cells were treated with MEL and ATRA at specified doses. Then, 24 h after the treatments, resazurin (Sigma-Aldrich) at a final concentration of 100 µg/mL was added into each well, and the cells were incubated for 4 h at 37 °C. Treatments were carried out in triplicate. Fluorescent intensity was measured by a microplate reader Infinite F200 (Tecan, Grodig, Austria) at an excitation wavelength of 535 nm and emission wavelength of 595 nm. The data are presented as the percentage of control cells (untreated samples).

### 4.4. Cell Cycle Analysis

After treatment, the cells (10^6^) were washed with PBS and fixed in 70% ethanol for 2 h at RT. The cells were again rinsed with PBS and resuspended in 500 µL of PBS containing 10 µg/mL of PI and 50 µg/mL of RNase. The samples were kept in the dark at RT for 30 min and analyzed by an Accuri C6 flow cytometry (BD Bioscience, San Jose, CA, USA). The distribution of cells in different phases of the cell cycle was estimated by ModFit LT soft (Verity Software House, Topsham, ME, USA).

### 4.5. Determination of the Mitotic Index

Mitotic activity was determined, as follows: cells incubated for 96 h under different conditions were centrifuged (250× *g*, 10 min), resuspended in PBS, and fixed with 70% ethanol (30 min, RT). Then, fixed cells were stained with bisBenzimide H33342 (Sigma-Aldrich), and mitotic cells were calculated while using a DM 6000 fluorescent microscope (Leica, Germany). The mitotic index (MI) was determined from the formula MI = (P + M + A + T)/N, where P + M + A + T is the sum of all cells in phases: prophase, metaphase, anaphase, and telophase, respectively, and N is the total number of cells.

### 4.6. Cell Growth Assays

Cell growth was estimated by counting cells at different time points after treatment. Cells were collected by centrifugation for 10 min at 250× *g* and washed with PBS. Then, the cells were stained with 0.4% trypan blue (Sigma-Aldrich) to evaluate the cell number and cell viability. Three samples per group were counted in each experiment, and the experiments were performed at least in triplicate.

### 4.7. Electrophoresis and Western Blot Analysis

HL-60 cells (2 × 10^5^ cells/mL) treated with MEL and/or ATRA were harvested, washed with ice-cold PBS twice, and lysed in lysis buffer (50 mM Tris–HCl (pH 7.4), 150 mM NaCl, 1% Triton X-100, 0.1% SDS, 1 mM EDTA, 1 mM Na_3_VO_4_, and 1 mM NaF) supplemented with proteinase/phosphatase inhibitors. The extracts were incubated on ice for 30 min and then centrifuged at 13,000× *g* for 20 min at 4 °C The supernatants were collected and quantified for protein concentration by using the Bradford protein assay. Then, the supernatants were solubilized by 4× Laemmli sample buffer (Bio-Rad). To prepare samples for the determination of the level of OxPhos Complexes, the aliquots (2 mg/mL) of cell lysates were heated to 37 °C for 3 min. To determine the level of mitochondrial proteins, the samples were heated to 95 °С for 5 min and applied to the gel. Protein samples were separated by 12.5% SDS-PAGE and transferred to a nitrocellulose membrane at 300 mA for 1 h. The membrane was blocked in Roti-block solution for 1 h at room temperature then incubated with the primary antibody at 4 °C overnight and then with an HRP-conjugated secondary antibody. The monoclonal anti-CNPase antibody (anti-CNPase Ab) was obtained as described [57], the polyclonal TSPO antibody was from Abcam polyclonal Bcl-xL antybody was from Cell Signalling and the monoclonal Bcl-2 antibody was from Santa Cruz. Alterations in the level of subunits of ETC complexes were determined while using a Total Oxphos Rodent WB Antibody Cocktail (Abcam). The Oxphos Antibody Cocktail consists of complex V alpha subunit (CV-ATP5A-55 kDa), complex III core protein 2 (CIII-UQCRC2-48 kDa), complex IV subunit I (CIV-MTCO1-40 kDa), complex II subunit 30 (CII-SDHB-30 kDa), and complex I subunit NDUF88-20 kDa (CI-NDUFB8). The α-tubulin antibody (1:1000 dilution; Cell Signaling, Danvers, MA, USA) was used as a loading control. The blot was detected by an ECL detection system (ChemiDoc Touch Imaging System, Bio-Rad). Protein bands were quantified by densitometry (Image Lab program).

### 4.8. Statistical Analysis

All data were presented as means ± S.D. Statistical significance was verified by the Student′s *t*-test using the Sigmaplot software (Systat Software Inc., San Jose, CA, USA).
